# Prevalence of Dental Fluorosis Among Primary School Children in Rural Areas of Chidambaram Taluk, Cuddalore District, Tamil Nadu, India

**DOI:** 10.4103/0970-0218.42047

**Published:** 2008-07

**Authors:** S Saravanan, C Kalyani, MP Vijayarani, P Jayakodi, AJW Felix, S Nagarajan, P Arunmozhi, V Krishnan

**Affiliations:** Division of Community Dentistry, Rajah Muthiah Dental College and Hospital, Annamalai University, Chidambaram, Tamil Nadu, India; 1 Department of Chemistry, Rajah Muthiah Dental College and Hospital, Annamalai University, Chidambaram, Tamil Nadu, India; 2 Division of Periodontology, Rajah Muthiah Dental College and Hospital, Annamalai University, Chidambaram, Tamil Nadu, India

**Keywords:** Chidambaram taluk, community fluorosis index, Cuddalore district, dental fluorosis, water fluoride level

## Abstract

**Background:**

Fluorosis is one of the common but major emerging areas of research in the tropics. It is considered endemic in 17 states of India. However, the Cuddalore district of Tamil Nadu is categorised as a fluorosis non-endemic area. But clinical cases of dental fluorosis were reported in the field practice area of Department of Community Medicine, Rajah Muthiah Medical College, Annamalai University, Chidambaram. Since dental fluorosis has been described as a biomarker of exposure to fluoride, we assessed the prevalence and severity of dental fluorosis among primary school children in the service area.

**Materials and Methods:**

Children studying in six primary schools of six villages in the field practice area of Rural Health Centre of Faculty of Medicine, Annamalai University, Chidambaram, were surveyed. Every child was clinically examined at the school by calibrated examiners with Dean's fluorosis index recommended by WHO (1997). Chi-square test, Chi-square trend test and Spearman's rank correlation coefficient test were used for statistical analysis.

**Results:**

Five hundred and twenty-five 5- to 12-year-old school children (255 boys and 270 girls) were surveyed. The overall dental fluorosis prevalence was found to be 31.4% in our study sample. Dental fluorosis increased with age *P* < 0.001, whereas gender difference was not statistically significant. Aesthetically objectionable dental fluorosis was found in 2.1% of the sample. Villages Senjicherry, Keezhaperambai and Kanagarapattu revealed a community fluorosis index (CFI) score of 0.43, 0.54 and 0.54 with 5.6%, 4.8% and 1.4% of objectionable dental fluorosis, respectively. Correlation between water fluoride content and CFI values in four villages was noted to be positively significant.

**Conclusion:**

Three out of six villages studied were in ‘borderline’ public health significance (CFI score 0.4-0.6). A well-designed epidemiological investigation can be undertaken to evaluate the risk factors associated with the condition in the study region.

Fluorine, a member of the halogen family, is an element essential for normal growth, development and maintenance of human health. Fluoride plays an important role in preventive dentistry due to its cariostatic potential. However, excessive intake of fluoride leads to dental and skeletal fluorosis. India lies in a geographical fluoride belt, which extends from Turkey up to China and Japan through Iraq, Iran and Afghanistan; of the 85 million tons of fluoride deposits found on the earth's crust, nearly 12 million tons are in India.(1,2) Consequently, fluorosis is an endemic condition prevalent in 17 states of India.(3)

In Tamil Nadu, fluorosis has been reported to be endemic in the districts of Dharmapuri, Erode, Salem, Coimbatore, Trichy, Madurai, Vellore and Virudhunagar.(3,4) However, in our study area, -the field practice area of the Department of Community Medicine, Rajah Muthiah Medical College and Hospital (RMMC and H), Chidambaram, Cuddalore district, which is a non-endemic area, clinical cases of dental fluorosis were reported when we assessed caries prevalence and treatment needs among primary school children. Clinical dental fluorosis being the most convenient biomarker of fluoride exposure(1) evoked the thought of conducting the present study with the following objectives:

1. To assess the prevalence and severity of dental fluorosis among primary school children in the study area.
2. To calculate the community fluorosis index (CFI) in the study population.
3. To provide baseline data and information about dental fluorosis to public health authorities for planning appropriate preventive strategies.

## Materials and Methods

This cross-sectional study was conducted among primary school children aged 5 to 12 years from classes 1 to 5 in the rural areas of Chidambaram taluk, Cuddalore district, Tamil Nadu.

### Study area

The Rural Health Centre of RMMCandH, Annamalai University, Chidambaram, decided on the study area. The Rural Health Centre is located at South Pichavaram, 12 km east of Chidambaram town and 4-6 km from the Bay of Bengal coast covering six villages namely (1) South Pichavaram, (2) Kanagarapattu, (3) T.S. Pettai, (4) North Pichavaram, (5) Keezhaperambai and (6) Senjicherry.

The study site has a land area of 16.4 km^2^ with a total population of 6089. The study area is a tropical dry and wet region, which is wholly an agricultural belt except T.S. Pettai, where fishing is one of the main occupations. The majority of people here are from lower socio-economic class with an average family annual income of Rs. 12,000 except for T.S. Pettai, where the income ranges between Rs. 12,000 and Rs. 18,000. There are six primary schools and one high school, where 99% of children of 5-10 years age group and around 85% of children of 11-16 years age group attend schools (Source: Village Administrative Officer).

### Study population

All the primary schools located in the service area of RMMCandH were chosen as research setting. The coverage of children was confined only to primary schools as each village had a primary school and 99% of the children of primary school age group in the study area were attending schools. High school children were not included as only 85% of the children of high school age group (11-16 years) in the study area were attending schools. Moreover, children from other villages were also attending this high school. All the children present in these six primary schools formed the study population. Hence the study population comprised 531 primary school children. It was confirmed that all the children were continuous residents of the study area since birth.

### Clinical examination

Written consent from parents and approval from concerned school authorities were obtained. The survey was conducted from July 2003 to March 2004. Oral examination was performed by three trained and calibrated dentists (SS, CK and MPV). The presence and severity of dental fluorosis was recorded using Dean's index (1942) according to WHO (1997) criteria.(5) Each tooth in the mouth was rated according to one of the six categories of Dean's index, and the individual's dental fluorosis score was arrived at based on the severest form recorded for two or more teeth. CFI(6) was calculated to identify villages where dental fluorosis has been a common public health problem. CFI was computed by summing up the scores of individual grades of dental fluorosis as described by Dean and dividing the sum by the total sample size. The public health significance of CFI values was as below:

CFI value rangePublic health significance0.0-0.4Negative0.4-0.6Borderline0.6-1.0Slight1.0-2.0Medium2.0-3.0Marked3.0-4.0Very marked

### Training and calibration of examiners

Prior to the survey, the examiners participated in a two-day training and clinical calibration exercise. Forty-eight school children were examined by each of the three investigators to assess inter-examiner reliability. Intra-examiner reproducibility was assessed by re-examining 10% of the sample. At the end of each day during the course of survey, 10 children were re-examined by each examiner to maintain intra-examiner consistency.

### Sources of drinking water

Five out of six villages studied had a common public drinking water supply system. In this scheme, water was pumped from the bore well into a storage tank erected at the central location of each village and taps were provided near the tanks. Water was also distributed through a pipe network and taps provided in the streets. In one village, South Pichavaram alone, the sources of drinking water were a common well and a hand pump closely connected to the well.

### Fluoride estimation in drinking water

Water samples were collected in 500-ml plastic bottles, which were doubly rinsed with distilled water. They were labeled, coded and sent to laboratory for fluoride estimation on the same day. Fluoride analysis was done at the Chemistry Department, Annamalai University, using SPADNS method.(7) Duplicate samples were taken after a month to confirm the fluoride level.

### Statistical analysis

The association of dental fluorosis with gender as well as with age was studied using Chi-square test and Chi-square trend test, respectively. Spearman's rank correlation coefficient test was used to measure the correlation between water fluoride level and CFI.

## Results

Among 531 primary school children, 525 were included in the study as the remaining six children were chronic absentees. Gender distribution in the sample was 255 (48.6%) boys and 270 (51.4%) girls.

Overall, 31.4% of the sample showed some grades of dental fluorosis. Dental fluorosis was more prevalent among boys than girls. However, gender difference was not statistically significant (*P* > 0.05, N.S.). The prevalence of dental fluorosis was found to increase with age (*P* < 0.001) [Table 1].

**Table 1:** Prevalence of dental fluorosis according to gender and age

Variables	Number of children		Prevalence (%)
		
	Examined	Affected	
Gender*			
Boys	255	86	33.7
Girls	270	79	29.3
Age group†			
5-6	181	39	21.5
7-8	198	71	35.9
9-10	129	49	38
11-12	17	6	35.3
Total	525	165	31.4

* X^2^ = 1.23, d.f. = 1, *P* > 0.05 (N.S.)

† Chi-square trend = 9.41, d.f. = 1, *P* < 0.001

Table 2 depicts the prevalence and severity of dental fluorosis along with CFI values in different villages. The villages Senjicherry, Kanagarapattu and Keezhaperambai recorded higher prevalence and higher CFI scores. Moderate and severe grades of fluorosis (aesthetically objectionable dental fluorosis) were noticed in 2.1% of the sample.

**Table 2:** Prevalence and severity of dental fluorosis by study area with community fluorosis index values

Villages (Subjects)	Prevalence		Severity of dental fluorosis *n* (%)						CFI
		
	*n*	%	Normal	Questionable	Very mild	Mild	Moderate*	Severe*	
North Pichavaram (77)	22	28.6	55 (71.4)	19 (24.7)	3 (3.9)	-	-	-	0.16
T.S. Pettai (130)	27	20.8	103 (79.2)	16 (12.3)	8 (6.2)	2 (1.5)	-	1 (0.8)	0.18
South Pichavaram (115)	29	25.2	86 (74.8)	12 (10.4)	13 (11.3)	2 (1.7)	-	2 (1.7)	0.27
Senjicherry (71)	26	36.6	45 (63.4)	15 (21.1)	4 (5.6)	3 (4.2)	3 (4.2)	1 (1.4)	0.43
Kanagarapattu (70)	32	45.7	38 (54.3)	11 (15.7)	11 (15.7)	9 (12.9)	1 (1.4)	-	0.54
Keezhaperambai (62)	29	46.8	33 (53.2)	9 (14.5)	14 (22.6)	3 (4.8)	3 (4.8)	-	0.54
Total (525)	165	31.4	360 (68.6)	82 (15.6)	53 (10.1)	19 (3.6)	7 (1.3)	4 (0.8)	0.32

* Objectionable dental fluorosis

Table 3 depicts fluoride concentration in drinking water, CFI values and percentages of objectionable dental fluorosis in various villages. Keezhaperambai and Kanagarapattu recorded lower ppm levels (< 0.1 ppm). On enquiring the local inhabitants and the Village Administrative Officer, we came to understand that the water sources in the above two villages were changed before two to three years of the study. This could be the reason why such a low ppm was recorded in the above two villages. In the remaining four villages, the water supply was constant for 10-12 years. Hence, the degree of correlation between water fluoride level and CFI values was measured only for those four villages. A highly positive significant correlation was found between them (*P* < 0.001).

**Table 3:** Distribution of water fluoride concentration, community fluorosis index values and objectionable dental fluorosis according to study area

Villages	Fluoride concentration in drinking water (ppm)	Community fluorosis index value	Objectionable dental fluorosis (%)
North Pichavaram	0.25†	0.16†	0.00
T.S. Pettai	0.56†	0.18†	0.77
South Pichavaram	0.66†	0.27†	1.74
Senjicherry	0.67†	0.43†	5.63
Kanagarapattu	<0.1	0.54	1.42
Keezhaperambai	<0.1	0.54	4.84

† rho = 0.99; *P* < 0.001

### Inter-examiner consistency

Inter-examiner consistency for the presence or absence of dental fluorosis was excellent (100%). However, the agreement regarding the degrees of fluorosis between SS and CK, SS and MPV and CK and MPV was 83.33%, 75% and 75%, respectively.

### Intra-examiner reproducibility

For the presence or absence of dental fluorosis, the intra-examiner reproducibility was excellent (100%). However, for the degrees of fluorosis, the agreement between the examinations by SS, CK and MPV was 100%, 60% and 80%, respectively.

## Discussion

Fluorosis is endemic in almost two-third states in India. Excess fluoride in groundwater is mainly the key factor. One in 10 villages of Rajasthan has excessive content of fluoride in its water supply.(8) About 62 million people are at risk of developing fluorosis from drinking high-fluoride ion water in India. Six million children below the age of 14 years are affected.(9) Dental fluorosis is endemic in 150,000 villages in India.(10)

In the present study, nearly one-third of children had experienced dental fluorosis of 31.4%. This estimate is very close to the findings (29.35%) in rural school children in Lucknow district, Uttar Pradesh.(11) However, a higher prevalence of 92.73% was recorded among school children of similar age group in the village of Juai Kalan, Bhiwani district, Haryana.(12) On the other hand, a lower prevalence of 16.8% was recorded in rural school children in Kerala.(13)

Not surprisingly, in the present study, dental fluorosis revealed no significant difference between genders. This is consistent with other studies conducted among rural school children in Haryana(14) and Karnataka.(15) Similar findings were recorded in rural Tanzania.(16) However, in Kerala, a higher prevalence among girls was reported.(13)

In this study, the prevalence of dental fluorosis was found to increase with age. This trend is consistent with the findings of DCI among children in rural Tamil Nadu.(17) Similar finding was documented among rural school children in Haryana.(12) One possible reason is that most of the teeth in the 5-6-year age group are deciduous (primary teeth), and much of the mineralization process occurs in the intra-uterine phase, where the placenta serves as a partial barrier to the transfer of fluoride to the developing primary teeth.(18) Other reasons for lower prevalence in the younger age groups may be that (i) the period of enamel formation for primary teeth is shorter and hence the exposure to fluoride is shorter;(19) (ii) the enamel of primary teeth is thinner than that of permanent teeth(19) and (iii) the rapidly growing skeleton of foetus may absorb fluoride at more rapid rate since fluoride is a hard-tissue seeker and is thus less available for primary teeth.(20) On the other hand, the greater body size and weight, the increased physical activity and the kind of food consumed may lead to a higher water intake and thus a higher prevalence in older age groups.(11)

Our study reports higher CFI values of 0.43, 0.54 and 0.54 in Senjicherry, Keezhaperambai and Kanagarapattu villages, respectively, indicating a ‘borderline’ (CFI score 0.4-0.6)(6) public health significance at one end and a ‘negative’ (CFI score less than 0.4)(6) public health significance in the villages North Pichavaram, T.S. Pettai and South Pichavaram, with lower CFI values of 0.16, 0.18 and 0.27, respectively, on the other end as per Dean's (1946) classification.(6)

In the present study, we observed a highly positive significant correlation between fluoride ion concentration in water and CFI scores, i.e., as the fluoride level in water increases, the CFI value also increases indicating the increase in the percentage of objectionable dental fluorosis. This is in accordance with the previous findings.(15)

Galagan and Lamson(21) reported 0% objectionable dental fluorosis at 0.5 ppm of fluoride in drinking water with a CFI score of 0.3 at a mean annual temperature of 21°C (70°F) in Arizona communities. According to Dean, the threshold of objectionable dental fluorosis lies between a CFI value of 0.4 and 0.6.(6) However, the present study shows 0.8% and 1.7% of objectionable dental fluorosis at 0.56 ppm (CFI value = 0.18) and 0.66 ppm (CFI value = 0.27), respectively, at a mean maximum annual temperature of 36.3°C (97.3°F).(22) This clearly indicates that even at a minimal fluoride concentration of 0.56 ppm in drinking water, with a CFI score of 0.18, unacceptable dental fluorosis was noticed due to considerable intake of water (resulting in higher fluoride ingestion) following a higher daily temperature. In addition, definite dental fluorosis had been noticed in significant number of children in Lucknow even at a fluoride level of 0.4 ppm.(11) Hence a CFI value of less than 0.4 - a ‘negative’ public health significance as described by Dean(6) - may not be appropriate for our climatic condition.

Thus, the ‘upper’ permissible limit of fluoride in drinking water has to be calculated based on temperature so as to prevent the occurrence of objectionable dental fluorosis in the study area. However, some additional factors were found to be of importance in the development of dental fluorosis such as the consumption of fish (as these were coastal areas) and tea, the nutritional status of individuals, the environmental factors, nature of dentifrices used and average water intake, which requires further investigation.

### Limitations of the study

The present study reports dental fluorosis in the mixed dentition on examining primary school children. However, reporting fluorosis in mixed dentition is not very straightforward. Data about dental fluorosis on permanent dentition (i.e., high school children) is more appropriate. However, information furnished in the present study can be utilized as preliminary data.

### Implications of the present study

Cuddalore district in Tamil Nadu is categorized as a fluorosis non-endemic area. As per the findings of the present study, it is evident that three villages in the study area are in the ‘borderline’ category of public health significance. Our study underscores the need for conducting detailed fluoride mapping and geochemical surveys of existing water sources of Cuddalore district. Nagarajan *et al.*(23) reported a maximum concentration of 1.2 ppm of fluoride in groundwater samples collected in and around Chidambaram town (12 km from the study area). Moreover, groundwater samples collected at different locations in and around Cuddalore SIPCOT area (around 40 km from the study area) showed up to a maximum concentration of 2.6 ppm of fluoride.(24) Hence periodic surveys are needed to monitor dental fluorosis and to conduct routine water analyses to identify the high-risk communities.

## Conclusion

The present study acts as a pointer to public health physicians, dentists, chemists, planners, administrators, engineers and water supply authorities. The information furnished can be utilized as preliminary data, and a well-designed epidemiological investigation can be undertaken at taluk level and district level to confirm and assess dental fluorosis and to evaluate the risk factors associated with the condition in the study region.
